# Green Approach to Enhance the Recovery of Polyphenols from Blackcurrant and Bilberry Leaves: Evaluation of Microwave-Assisted and Pressurized Liquid Extraction

**DOI:** 10.3390/molecules29061351

**Published:** 2024-03-18

**Authors:** Ivona Elez Garofulić, Maja Repajić, Ena Cegledi, Erika Dobroslavić, Ana Dobrinčić, Zoran Zorić, Sandra Pedisić, Tatjana Franković, Martina Breški, Verica Dragović-Uzelac

**Affiliations:** 1 Faculty of Food Technology and Biotechnology, University of Zagreb, Pierotti Street 6, 10000 Zagreb, Croatia; ivona.elez@pbf.unizg.hr (I.E.G.); ecegledi@pbf.hr (E.C.); edobroslavic@pbf.hr (E.D.); adobrincic@pbf.hr (A.D.); spedisic@pbf.hr (S.P.); tatjana.dutkovic@gmail.com (T.F.); ma.brele@gmail.com (M.B.); vdragov@pbf.hr (V.D.-U.); 2 Department of Ecology, Agronomy and Aquaculture, University of Zadar, P. Kasandrića 3, 23000 Zadar, Croatia; zzoric@unizd.hr

**Keywords:** blackcurrant leaves, bilberry leaves, antioxidant, polyphenols, extraction

## Abstract

The aim of the present study was to evaluate microwave-assisted (MAE) and pressurized liquid extraction (PLE) for the recovery of polyphenols from blackcurrant and bilberry leaves and the preservation of their antioxidant activity. The extractions were carried out varying the solvent/solid (SS) ratio, temperature and time. During MAE, increasing the SS ratio increased the polyphenol concentration in the extracts from blackcurrant and bilberry leaves, while increasing the temperature had a positive effect only on bilberry polyphenols. During PLE, only a temperature increase was a determining factor for the isolation of blackcurrant leave polyphenols. Based on polyphenol recovery, optimal extraction parameters were established resulting in a yield of 62.10 and 56.06 mg/g dw in the blackcurrant and bilberry MAE extracts and 78.90 and 70.55 mg/g dw in the PLE extracts. The optimized extracts were profiled by UPLC ESI MS^2^, and their antioxidant capacity was evaluated through FRAP, DPPH, ABTS and ORAC assays. The characterization of the extracts by UPLC ESI MS^2^ confirmed flavonols as the predominant compounds in both blackcurrant and bilberry leaves, while flavan-3-ols and procyanidins were the main compounds responsible for high antioxidant capacity as confirmed by the ABTS and ORAC assays. Due to the extract composition and antioxidant capacity, PLE proved to be a technique of choice for the production of blackcurrant and bilberry leave extracts with high potential for use as value-added ingredients in the food and nutraceutical industry.

## 1. Introduction

Blackcurrant (*Ribes nigrum* L.) and bilberry (*Vaccinium myrtillus* L.) are two berry species consummated fresh or processed into various products worldwide, not only for their nutritional value but also for the numerous health benefits arising from their phytochemical composition. Thereafter, both species are considered “superfood”, i.e., food with an exceptionally high content of bioactives that are responsible for its health-promoting properties [1]. In addition to vitamins and fiber, these properties are mainly attributed to polyphenols, which comprise phenolic acids and flavonoids, including anthocyanins as the ones responsible for the fruit color. All of these compounds have been proven to possess antioxidant, antimicrobial and anti-inflammatory effects, which are responsible for the numerous health benefits related to the consumption of both blackcurrant and bilberry. However, it is not only the fruits of these two types of berry that are rich in polyphenols; the leaves of blackcurrant and bilberry also have a similar or even richer phenolic composition [1]. Blackcurrant leaves are rich in chlorogenic acid and its isomers [2], quercetin and kaempferol glycosides and flavan-3-ols such as catechin, epicatechin and epigallocatechin [3]. Bilberry leaves are also a source of hydrocinnamic acids [4], proanthocyanidins and quercetin and kaempferol derivatives [5]. The health-promoting properties attributed to these substances are related to their polyphenolic composition. Hence, the leaves of blackcurrant are associated with significant antioxidant and anti-inflammatory properties and have also been used for the treatment of diarrhea and cough [1], while bilberry leaves are known for their antistaphylococcal and anticancer activity [6,7]. Unfortunately, despite their known bioactive potential, a large amount of the leaves is often discarded during fruit harvesting.

The starting point in the utilization of blackcurrant and bilberry leaves’ benefits for future product design is the isolation of their polyphenols. Nowadays, in compliance with the green technology concept, advanced extraction techniques have taken priority over the conventional methods, primarily in terms of time and energy savings. Among these, microwave-assisted extraction (MAE) and pressurized liquid extraction (PLE) have been widely applied for the isolation of polyphenols from plant matrices, including leaves [8,9,10,11]. The MAE extraction principle is based on the interaction of microwaves with polar compounds through ionic conduction and dipole rotation which cause heating of the sample by friction and consequently cell wall disruption, thus enabling a better solvent penetration into the matrix and a more efficient extraction of the target compounds [12,13]. The PLE mechanism is based on the application of high temperature and pressure, which keeps the extraction solvent in a liquid state throughout the process, thus improving the solubility and extraction of the target compounds [14]. Although based on different mechanisms, both techniques provide a fast, effective and sustainable approach for the extraction of plant polyphenols. In order to achieve the maximal output of these techniques, both the extraction parameters and the plant characteristics must be taken into account. The efficiency of MAE is primarily determined by the selection of optimal temperature, irradiation time and microwave power, while PLE can be managed by operating the temperature, static extraction time and number of extraction cycles while maintaining high pressure. The elevation of the temperature as well as the prolongation of the extraction time generally lead to increased diffusion rate and better solubility of the target compounds from a plant matrix, but must be evaluated and selected with regard to the possible degradation of sensitive compounds [15,16]. In MAE, the microwave power affects the extraction yield through increased molecular interactions in the electromagnetic field and is strongly related to the extraction temperature elevation [17].

For both techniques, the solvent-to-solid ratio is an important parameter affecting the polyphenolic yield in terms of change in solubility and extraction equilibrium constant. Namely, a higher amount of extraction solvent promotes the extraction yield through an increase in the concentration gradient and, consequently, the diffusion rate between the plant material and the extraction solvent until equilibrium is reached [18,19].

Additionally, not one of the mentioned parameters can be universally established without considering the characteristics of the plant material itself, in particular the nature of the present targeted polyphenols. Therefore, the aim of this study was to exploit the antioxidant potential of blackcurrant and bilberry leaves through the evaluation of two green extraction techniques, MAE and PLE, for the isolation of polyphenols, followed by a detailed characterization of the obtained extracts in terms of their composition and antioxidant capacity.

## 2. Results and Discussion

### 2.1. The Effect of MAE and PLE Parameters on the Total Phenolic Yield from Blackcurrant and Bilberry Leaves

To evaluate the effect of two green extraction techniques, MAE and PLE, on the total phenolic content (TPC) of blackcurrant and bilberry leaves, extractions were carried out using a 30% ethanol solution at different settings of temperature, extraction time and solvent/sample (SS) ratio., We used extraction temperatures of 60, 70 and 80 °C for MAE and of 100, 125 and 150 °C for PLE. Irradiation time for MAE and static extraction time for PLE were varied between 5 and 10 min, while the SS ratio was set to 20, 30 and 40 mL/g. The results of TPC determination in all experimental trials are shown in Table 1.

The TPC in blackcurrant leaves ranged from 49.45 to 73.26 mg/g dry weight (dw) in MAE extracts and from 42.92 to 78.90 mg/g dw in PLE extracts. The ranges obtained were similar for both techniques, but it should be noted that PLE produced more extracts with phenolic yield at the upper end of the mentioned range. These values were slightly higher than the TPC of 39.96 mg/g dw in blackcurrant leaves extracted by ultrasound-assisted extraction, as reported by Nour et al. [20], and that of 23.08 mg/g dry sample obtained by MAE, as reported by Cao-Ngoc et al. [21], while they are in line with the TPC of 70.57 mg/g reported by Ziemlewska et al. [22] for extracts produced by ultrasound-assisted extraction. The TPC range in bilberry leaves in this study, specifically, 33.74–70.55 mg/g dw, was slightly higher than the TPC of 45.18 mg/g dw reported by Stanoeva et al. [23], while it was in line with or lower than the range of 54.7–106.9 mg/g dw, depending on the harvest year and vegetation period, reported by Bujor et al. [24].

The results showing the effect of the MAE and PLE extraction parameters on the TPC of blackcurrant and bilberry leaves are shown in Table 2.

In the case of MAE application for the isolation of phenols from blackcurrant leaves, only the SS ratio showed to be a significant parameter, while the effects of temperature and irradiation time were not statistically significant. For bilberry leaves, in addition to the SS ratio, temperature was also a significant factor during the extraction. In this study, temperature was selected as a controllable parameter, meaning that the constant microwave power of 400 W was applied only in respective periods of time required to maintain the set temperature. As can be seen from the results, the highest applied temperature of 80 °C was optimal for both blackcurrant and bilberry leaves’ phenol isolation. A similar conclusion was made by Dobroslavić et al. [9] for the MAE of *Laurus nobilis* L. leaves. Although the irradiation time was not a significant factor for MAE, some differences were observed between blackcurrant and bilberry. During the extraction of blackcurrant phenols, no difference was observed between 5 and 10 min of exposure to microwaves, while a 10 min exposure, on the other hand, produced a slightly higher phenolic yield from bilberry leaves. Generally, a prolonged microwave exposure favors the extraction of phenols; however, if excessive, it can lead to the degradation of the phenolic compounds [25]. The SS ratio significantly affected the phenolic yield from both blackcurrant and bilberry leaves, leading to an increase with a higher amount of extraction solvent used. This trend is in an alignment with the general observation that an increase in solvent volume positively affects the extraction efficiency through the acceleration of the mass transfer between the solvent and the material and the promotion of the solubility of the target compounds [26]. The increase in the concentration gradient between the samples and the solvent in this study benefited the phenolic yield, thereby resulting in the highest TPC when the highest SS ratio of 40 mL/g was applied. A positive effect of higher solvent volume for the isolation of phenols during MAE was also observed for grapevine and strawberry leaves [27,28], where SS ratios of 40 and 60 mL/g were set as optimal for maximal recovery. Regarding the variation in TPC between samples, it can be observed that the mean values obtained in different MAE conditions were higher for blackcurrant leaves than for bilberry leaves, indicating that blackcurrant leaves are a richer source of phenolics.

The mean TPC values in the blackcurrant and bilberry extracts obtained with PLE were slightly higher than those obtained with MAE. The higher effectiveness of PLE can be attributed to the application of high pressure during the extraction process. Specifically, high pressure allows for a better penetration of the solvent into the plant matrix and delays the formation of air bubbles in the matrix, which prevent the solvent to thoroughly reach the target compounds [29]. For blackcurrant leaves, temperature significantly affected the TPC, while static extraction time and SS ratio had no effect. In the case of bilberry leaves, none of the examined parameters in the proposed ranges had a statistically significant effect. A temperature increase during PLE favored the extraction yield from blackcurrant leaves, which reached the highest value at 150 °C, while for bilberry leaves, the highest phenolic yield was obtained at 125 °C, as a further increase had a negative effect. Generally, temperature is considered the main factor in extraction processes, as it regulates the solubility of the target compounds in the extraction solvent through varying the molecular diffusivity and viscosity of the solvent [30,31]. Similar temperatures were found to be optimal for the PLE of polyphenols from strawberry leaves [10], *Stevia rebaudiana* leaves’ phenolic acids [32] and *L. nobilis* leaves’ polyphenols [8].

In this study, only a variation in static extraction time between 5 and 10 min was evaluated, while all PLE extractions were performed in three consecutive extraction cycles. As the results indicate, no significant difference was observed in terms of PLE duration, although there was a slightly higher phenolic yield from bilberry leaves after 10 min of extraction. This observation may be related to the delivery of fresh solvent within each extraction cycle, which prevented the saturation of the solvent with the target compounds [33]. The static extraction time range between 5 and 10 min was also reported to be optimal for chaga fungus phenolics, showing a constant increase in phenolic yield in single-factor experiments of PLE from 1 to 7 min [34]. Similarly, the optimal static extraction time for the extraction of *L. nobilis* leaves was 5 min within one extraction cycle [8], while for nettle leaves, it was 10 min during three extraction cycles [35]. Unlike what observed for MAE, the SS ratio was not a significant factor for PLE. The reason can again be found within the application of multiple extraction cycles and the supply of fresh solvent at each cycle, which minimized the effect of solvent saturation and extraction equilibrium. Although not significant, some differences can be outlined. During blackcurrant leave extraction, a slight decrease in TPC occurred when the SS ratio was increased from 30 to 40 mL/g, indicating the possible reaching of the extraction equilibrium. On the other hand, the TPC in bilberry leaves was the highest at the highest applied SS ratio of 40 mL/g. When the same PLE study design was applied for the isolation of strawberry leaves’ phenols, a similar effect was observed [10].

Based on the made observations, optimal conditions were established for the extraction of blackcurrant and bilberry leaves’ polyphenols, as follows: for MAE, 80 °C/5 min/40 mL/g for blackcurrant leaves and 80 °C/10 min/40 mL/g for bilberry leaves, corresponding to the TPC values of 62.10 and 56.06 mg/g dw, respectively; for PLE, 150 °C/5 min/30 mL/g for blackcurrant leaves and 125 °C/10 min/40 mL/g for bilberry leaves, corresponding to the TPC values of 78.90 and 70.55 mg/g dw.

### 2.2. Profiling of the Optimized MAE and PLE Extracts of Blackcurrant and Bilberry Leaves

The optimized MAE and PLE extracts of blackcurrant leaves were analyzed by UPLC ESI MS^2^, and the results of their polyphenolic composition are shown in Table 3.

A total of 47 polyphenols were identified in the blackcurrant extracts, including 14 phenolic acids, 21 flavanols, 4 flavan-3-ols, 5 flavones and 3 procyanidins. Their fragmentation patterns were reported previously [9,36,37,38]. Among the phenolic acids, chlorogenic, caffeic, gallic and *p*-hydroxybenzoic acids were present in the highest concentrations. The predominance of chlorogenic and caffeic acids in blackcurrant leaves was confirmed by Vagiri et al. [2] and Raudsepp et al. [3], while some studies, opposite to our observations, reported quinic [39], *p*-coumaric [20] and salicylic acid [40] as the main representatives. The concentrations of chlorogenic and caffeic acid in this study were significantly higher than 5.9–8.31 µg/g dw and 88.86 µg/g dw, respectively, reported by Nour et al. [20] and than 20.9 µg/g dw for chlorogenic acid and 40.6 µg/g dw for caffeic acid reported by Chrzanowski et al. [40]. On the contrary, Raudsepp et al. [3] determined chlorogenic acid in significantly higher concentration, reaching even 14.93 mg/g dry leaves. Flavonols were found to be the predominant group of polyphenols in blackcurrant leaves in this study, with high proportions of quercetin, kaempferol, their glucosides and quercetin rutinoside. The presence of quercetin, quercetin-3-rutinoside and myricetin as representatives of flavonols in blackcurrant leaves was confirmed by Nour et al. [20] and Chrzanowski et al. [40], although in significantly lower concentrations. In line with our characterization, Vagiri et al. identified quercetin-3-*O*-glucoside, kaempferol-3-*O*-rutinoside and kaempferol-3-*O*-glucoside, and Oszmianski et al. [41] and Vagiri et al. [42] isorhamnetin-3-*O*-rutinoside, isorhamnetin-3-*O*-glucoside and kaempferol acetylglucoside. Catechin and epicatechin were determined to be the major flavan-3-ols in blackcurrant leaves, similar to the reports of Raudsepp et al. [3] and Cyboran et al. [43]. This study also confirmed the presence of luteolin as the major flavone in blackcurrant leaves, as well as the presence of three procyanidins, with procyanidin B1 as the most abundant. To the extent of our knowledge, the individual characterization of flavones and procyanidins in blackcurrant leaves has not been reported previously; thus, this is the first report.

When observing the effect of the extraction technique, it can be concluded that PLE was more effective than MAE for the isolation of blackcurrant phenolic acids, in particular for quinic, 3-*p*-coumaroylquinic, chlorogenic, syringic, galloylquinic, gallic and *p*-coumaric acid, while 3,5-dicaffeoylquinic and ferruloylquinic acid were only identified in the PLE extract. The only exception was *p*-hydroxybenzoic acid. Its higher recovery from MAE could be related to the general principle regulating the stability of phenolic acids during MAE, specifically the claim that stability under microwave irradiation is higher for compounds with fewer substituents in the structure of the aromatic ring [44]. A similar trend could be observed for flavonoids and procyanidins. PLE was more effective for almost all flavonol representatives, except myricetin, quercetin and myricetin rhamnoside and arabinoside, noting that the differences in extraction efficiency in favor of MAE were not as pronounced. Additionally, the PLE extract had significantly higher amounts of luteolin and catechin, the main representatives of their respective phenolic classes, as well as a higher proportion of procyanidin B1. However, apigenin, luteolin glucoside and procyanidin B2, although in low concentrations, were only detected in the MAE extracts. On the other hand, the PLE extract contained quercetin and kaempferol pentoside, kaempferol acetylhexoside, quercetin pentosylhexoside, apigenin deoxyhexosyl hexoside, luteolin rutinoside and procyanidin trimer, which were not detected in blackcurrant leaves subjected to MAE. Although flavonoids and procyanidins would be expected to be more susceptible to thermal degradation due to their more complex structure than phenolic acids, PLE showed to be more effective than MAE for the isolation of all observed phenolic classes, despite the application of a significantly higher temperature than in MAE, namely, 150 versus 80 °C, resulting in an almost 3-fold higher concentration of total individual polyphenols. Flavonoids, being less polar than phenolic acids, may be more effectively isolated by PLE, regardless of the application of higher temperatures, which negatively affect them, as the pressure applied during PLE induces a reduction in solvent polarity and consequently promotes the solubility of less polar compounds [45]. Moreover, the pressure application decreases the tension between the sample and the solvent, and compounds with more hydroxyl substituents increase the amount of hydrogen bonds with the solvent, which enhances their solubility [45,46]. These results are in accordance with those reported by Terpinc et al. [10] for the comparison of MAE and PLE effects on individual polyphenols in strawberry leaves, where PLE was shown to be more effective than MAE for the isolation of flavonols, flavan-3-ols, flavones and procyanidins. Similarly, PLE provided a 2-fold higher polyphenolic yield from *Moringa oleifera* leaves than MAE and was proven to be a more effective technique for the isolation of phenolic compounds with a higher number of hydroxyl-type substituents and those sensitive to high temperatures [47]. In the same research, MAE allowed for a better recovery of quercetin and kaempferol, which is partially in line with our findings, as for blackcurrant leaves, MAE only benefited the extraction yield of quercetin.

The polyphenolic composition of the optimized MAE and PLE extracts from bilberry leaves is shown in Table 4.

Bilberry extracts comprised a total of 46 compounds, including 11 phenolic acids, 26 flavonols, 4 flavan-3-ols, 3 flavones and 2 procyanidins. The predominant phenolic acids were caffeic, chlorogenic, gallic and syringic acid. The prevalence of chlorogenic acid and its derivates in bilberry extracts was confirmed previously [4,5,41], as well as the presence of a significant amount of caffeic acid [4]. However, there are variations in reports regarding the dominant phenolic acid. Our results showed that syringic acid was present in the highest concentration in the MAE extract, while caffeic acid was the predominant compound in the PLE extract. Değirmencioğlu et al. [48] identified syringic acid as the most abundant compound in the leaves of bilberry from Turkey, Stefanescu et al. [49] reported the predominance of feruloylquinic acid in extracts of wild bilberry from Romania, while Brezoiu et al. [50] pointed out chlorogenic acid as the dominant compound in ethanolic bilberry extracts. The same authors identified caffeic acid only in hydroethanolic extracts, explaining its presence as a result of chlorogenic acid hydrolysis.

The predominant phenolic class in bilberry leaves determined in this study was that of flavonols, mainly represented by quercetin, kaempferol and their glycosides. In the MAE extract, the kaempferol aglycone was found in the highest concentration, while in the PLE extract the highest concentration was found for the quercetin glucoside. Similar to our results, although a lower amount was detected, Stanoeva et al. [23] reported the quercetin glucoside concentration of 236 mg/100 g dw as the highest among those of flavonols in Macedonian bilberry. The differences in the obtained concentrations could be attributed to the extraction technique, the solvent used or the origin of the plant material. Besides quercetin and kaempferol glycosides, our results indicated the presence of myricetin glycosides as well, which were previously only reported for Turkish and Rovaniemi bilberry [48,51]. Regarding flavan-3-ols, the MAE extract was the most rich in catechin, while the PLE extract was rich in epicatechingallate, with both compounds confirmed previously as the most prevalent within their class by Stanoeva et al. [23]. Flavones were detected in bilberry leaves as luteolin, apigenin and luteolin rutinoside. Reports on their presence in bilberry leaves are scarce, as they are mostly found in very low concentration [52]. Regarding procyanidins, our results displayed the presence of procyanidin B1 as the most abundant and procyanidin trimer in low amount. These findings comply with reports on the prevalence of procyanidin B-type dimer and trimer compounds in bilberry leaves [49].

When observing the full polyphenolic profiles of the bilberry leave MAE and PLE extracts, differences appeared not only in the quantitative, but also in the qualitative composition, indicating the PLE technique as the one providing a greater diversity of compounds. PLE provided significantly higher amounts of both hydroxycinnamic and hydroxybenzoic acids, while MAE was more effective only in the recovery of syringic acid. This observation is not in line with the results of Terpinc et al. [10] for strawberry leaves, reporting that the syringic acid content was 63% higher in the PLE extract than in the MAE extract; however, it aligns with the phenolic acid stability principle during MAE, claiming a better stability of methoxyilates during exposure to microwaves [44]. Regarding flavonoids, PLE was more effective for the isolation of all identified compounds apart from myricetin arabinoside, catechin and procyanidin B1, which were better recovered by MAE. These results are in accordance with observations made on blackcurrant leaves and may be related to the pressure effect on the enhancement of the solubility of flavonoid compounds during PLE [45].

When comparing the two plant samples analyzed, blackcurrant and bilberry leaves, the yield of total polyphenols identified by UPLC ESI MS^2^ was similar for both techniques. The main phenolic acids in both plants were caffeic acid and chlorogenic acid, the main flavonols were quercetin and kaempferol aglycones, the main flavan-3-ols and procyanidins were catechin and procyanidin B1 and the main flavones were luteolin and its rutinoside. However, some special features can be deduced from their detailed composition. In comparison, blackcurrant leaves contained a higher total amount of phenolic acids, which accounted for about 15 and 8% of the total polyphenol content in the MAE and PLE extracts, while bilberry leaves had a phenolic acid content of 7 and 5% in the MAE and PLE extracts, respectively. Interestingly, syringic acid dominated in the MAE bilberry extract, whereas chlorogenic acid predominated in both the blackcurrant extracts and the PLE blueberry extracts. In terms of flavonoid content and composition, blackcurrant and bilberry leaves differed with regard to their flavonol profile and flavan-3-ol content. Blackcurrant leaves showed a higher proportion of flavan-3-ols and a higher amount of acylated flavonol glycosides, while the bilberry extracts had a higher concentration of myricetin glycosides. All these variations in the composition of the extracts could be related to differences in the preferred extraction conditions. For example, the significance of the temperature effect during MAE was only observed for the bilberry leaves, and that particular extract was characterized by syringic acid, which, as mentioned above, is a methoxylated phenolic acid with high stability under microwave exposure. On the other hand, blackcurrant leaves were preferentially processed at higher temperatures during PLE, which improved the solubility of flavonoid compounds under the applied pressure.

In addition, for both blackcurrant and bilberry leaves, regardless of the extraction technique, the yields obtained during extraction optimization, measured using the Folin–Ciocalteu method, were significantly higher than the yields resulting from the sum of the yields of all compounds identified using UPLC ESI MS^2^. This discrepancy may be related to the low selectivity of the Folin–Ciocalteu reagent towards polyphenols, as it can also react with other compounds such as polysaccharides, organic acids, sugars [53,54] and, most importantly, chlorophylls [55], which is of particular relevance for chlorophyll-rich material such as the leaves used in this study.

### 2.3. Antioxidant Capacity of the Optimized MAE and PLE Extracts of Blackcurrant and Bilberry Leaves

The obtained optimized blackcurrant and bilberry leave extracts were analyzed for their antioxidant capacity through four different assays in order to provide a reliable display of their properties (Table 5).

The antioxidant capacity of blackcurrant leaves was significantly affected by the extraction technique employed, regardless of the assay applied. According to the FRAP, ABTS and ORAC assays, the PLE extract had a significantly higher antioxidant capacity than the MAE extract, which is in line with its higher TPC and individual polyphenolic content. Oppositely, the DPPH assay showed a higher antioxidant capacity of the MAE extract, although it had a lower TPC content and a lower concentration of all individual polyphenolic subclasses. As discussed for the polyphenolic characterization of the extracts, MAE favored the extraction of a limited number of compounds, including myricetin and its glycosides and the quercetin aglycone. As these flavonoids are known as strong antioxidants, the stronger DPPH radical scavenging capacity observed could be assigned to the aforementioned compounds, but the results of a Pearson’s correlation analysis between antioxidant capacity and the content of individual polyphenolic classes (Table 6) showed no significance for the DPPH assay. Thus, this discrepancy could be attributed to the assay limitations, such as the non-linear reactivity of antioxidants with radicals [56]. As the DPPH assay is a mixed assay, based on both HAT and ET mechanisms, its kinetics is strongly affected by the solvent, because the release of H atoms is limited by the strength of the solvent’s hydrogen bonding to the polyphenolic hydroxyl groups and DPPH radical, leading to a fast reaction in methanol and significantly slower one in acetone and ethanol [56,57]. As our extracts were prepared in an aqueous ethanol solution, this exception of the DPPH assay values could be explained considering the premise above. Regarding the range of antioxidant capacity obtained with the different assays, our values are slightly higher than those of Teleszko and Wojdylo [58], who reported an antioxidant capacity for blackcurrant leaves of 329.1 and 191.6 µmol TE/g dw by the ABTS and FRAP assays, respectively, and than the DPPH value of 200.3 µmol TE/g dw reported by Katsube et al. [59]. On the other hand, Ziobroń et al. [60] reported significantly higher values obtained with the ABTS, DPPH and FRAP assays than most of the previously reported ones, reaching even 7877.82 µmol TE/g dw for the DPPH radical scavenging capacity of blackcurrant leaves, and attributed them to differences in the extraction process, as the authors carried out a 2 h extraction in a water bath shaker using acidified methanol.

In the case of the bilberry leaves extracts, the extraction technique influenced the antioxidant capacity determined by the FRAP, DPPH and ABTS assays, providing higher values when PLE was used, which is in line with the corresponding polyphenolic content, while there was no significant difference between the techniques as regards the ORAC values. Generally, the antioxidant capacity of bilberry leaves was slightly lower than the one of blackcurrant leaves, which is also consistent with their respective polyphenolic content. The literature reports an antioxidant capacity of bilberry leaves of 793.0 and 595.8 µmol TE/g dw obtained using the ABTS and FRAP assays [61], which is close to our values. The ORAC values of bilberry leaves extracts were approximately 1.5-fold higher than the value of 251.6 µmol TE/g fresh weight reported for the bilberry fruit [62]. When observing the contribution of total or individual polyphenolic classes to the antioxidant capacity of both blackcurrant and bilberry leaves extracts produced by MAE and PLE, it was shown that only flavan-3-ols and procyanidins had a significant and very strong positive correlation with the antioxidant capacity as determined by both ABTS and ORAC assays for flavan-3-ols and by only the ORAC assay for procyanidins. The share of flavan-3-ols in the total UPLC ESI MS^2^ polyphenolic content was 4.73 and 6.08% in the blackcurrant leave MAE and PLE extracts, and 4.14 and 1.18% in the bilberry leave MAE and PLE extracts. Procyanidins were 7.76 and 9.50% of the total blackcurrant polyphenols extracted by MAE and PLE, and 15.41 and 1.96% of the total polyphenols in bilberry MAE and PLE extracts. Although those proportions are relatively low, compared to those of the flavonols, amounting to more than 50% of the total polyphenolic content, their significant contribution to the antioxidant capacity is a result of their structural characteristics. Soobrattee et al. [63] reported the antioxidant capacity of reference polyphenolic compounds to decrease in the order procyanidin dimer > flavan-3-ols > flavonols > hydroxycinnamic acids > hydroxybenzoic acids. The highest capacity of procyanidins is attributed to their hydroxyl groups and conjugated double bonds, while for flavan-3-ols, the capacity is strongly dependent on the presence of the galloyl moiety in gallates, increasing the number of free hydroxyl groups. Furthermore, the dominance of flavan-3-ols over flavonols in terms of antioxidant capacity may be a result of different oxidation mechanisms, as flavan-3-ol oxidation results in the formation of semiquinons, which couple to form oligomeric compounds, thereby retaining the catechol and pyrogallol structures and preserving their scavenging ability. Flavonols, contrarily, form quinones, which are prone to the redox cycle and may act as pro-oxidants [64,65]. The observed significance of flavan-3-ol and procyanidin contribution to the antioxidant capacity of the extracts may explain the differences between the total polyphenolic content of the MAE and PLE extracts and their respective antioxidant capacity, especially in the case of bilberry leaves. The bilberry extract obtained by MAE was relatively poor in total polyphenols, with only 7.01 mg/g dw of total compounds identified by UPLC ESI MS^2^. However, its ORAC value did not differ significantly from that of the PLE extract, with 27.99 mg/g dw of UPLC ESI MS^2^ total polyphenols. Still, the MAE extract had a 2-fold higher content of procyanidins than the PLE extract, namely, 1.08 versus 0.55 mg/g dw, which made the procyanidin content share in the MAE extract higher than 15%. Therefore, despite the lower UPLC ESI MS^2^ total polyphenol content, the bilberry extract obtained by MAE presented the same antioxidant capacity as the one produced by PLE.

## 3. Materials and Methods

### 3.1. Plant Material

The samples of dry blackcurrant and bilberry leaves were bought from Suban Ltd. (Strmec, Croatia). The blackcurrant (*Ribes nigrum* L.) leaves originated from Poland and had the batch number 20251, while the bilberry (*Vaccinium myrtillus* L.) leaves were from Croatia and part of the batch with the number 22-0101. The samples were kept in their original paper bag packaging and stored in a dry and dark place. Prior to the extraction, the dry leaves were milled in an electric grinder (CM 3260, Grundig, Nuremberg, Germany) and analyzed for their moisture content [66]. The moisture content of the blackcurrant and bilberry leaves was 9.66 and 9.72%, respectively.

### 3.2. Chemicals and Reagents

Ethanol used for the extractions and analyses as well as methanol used for the analyses were HPLC-grade and were purchased from Gram-Mol Ltd. (Zagreb, Croatia). Hydrochloric acid (37%) and glacial acetic acid (99–100%) were from Carlo Erba (Val-de-Reuil, France). HPLC acetonitrile was purchased from J.T. Baker Chemicals (Deventer, The Netherlands), and formic acid (98–100%) from T.T.T. Ltd. (Sveta Nedjelja, Croatia). Distilled water was of Milli-Q quality (Millipore Corp., Bed-ford, NY, USA). Trolox (6-hydroxy-2,5,7,8-tetramethylchroman-2-carboxylic acid), 2,4,6-tris(2-pyridyl)-s-triazine, 2,2-diphenyl-1-picrylhydrazyl radical, 2,2-azinobis(3-ethylbenzothiazoline-6-sulfonic acid) and 2,20-azobis(2-amidinopropane)hydrochloride were purchased from Sigma-Aldrich (Steinheim, Germany). Anhydrous sodium carbonate (99.5%), sodium acetate trihydrate sodium phosphate (96%) and potassium persulfate (99%) were obtained from Kemika (Zagreb, Croatia), iron(III) chloride hexahydrate from Gram-Mol Ltd., sodium chloride and the Folin–Ciocalteu reagent from Merck (Darmstadt, Germany), and fluorescein sodium salt from Honeywell Riedel-de-Haën (Bucharest, Romania).

Commercial standards of gallic, caffeic, chlorogenic, rosmarinic, syringic, *p*-coumaric, ferullic acid, kaempferol-3-rutinoside, myricetin and quercetin-3-glucoside were purchased from Sigma-Aldrich, catechin, epicatechin, epigallocatechingallate, epicatechingallate, luteolin, apigenin from Extrasynthese (Genay, France), and quercetin-3-rutinoside from Acros Organics (Geel, Belgium).

### 3.3. Extractions

#### 3.3.1. MAE

MAE of phenolic compounds from blackcurrant and bilberry leaves was performed in a microwave reactor Ethos Easy (Milestone, Sorisole, Italy) with adjustable microwave power up to 1900 W, operating at 2450 MHz, with a 30% aqueous ethanol solution as the extraction solvent. In order to determine the optimal extraction conditions which would result in the highest phenolic yield, the parameters of temperature (60, 70 and 80 °C), irradiation time (5 and 10 min) and SS ratio (20, 30 and 40 mL/g) were varied during the experiment (Table 1). The general extraction parameters were set as follows: 3 min preheating time, 1 min ventilation and cooling after the extraction, stirring level of 50% and microwave power of 400 W.

For the extraction, a mass of the ground sample was weighed, transferred into the extraction vessel according to the experimental plan and homogenized in 40 mL of extraction solvent. Extraction was performed according to the experimental plan, and afterwards the samples were filtered into a 50 mL volumetric flask and made up to the volume with the extraction solvent. The prepared extracts were stored at −18 °C in a nitrogen gas atmosphere until analyzed.

#### 3.3.2. PLE

PLE of phenolic compounds from blackcurrant and bilberry leaves was performed on a ThermoScientific™ Dionex™ ASE™ 350 extractor (Thermo Fisher Scientific, Sunnyvale, CA, USA) according to the experimental design (Table 1) using a 30% aqueous ethanol solution as the extraction solvent. The constant extraction parameters were as follows: pressure 10.34 MPa, nitrogen purge 30 s, flush volume 30%, and number of extraction cycles 3. The varying parameters were temperature (100, 125, and 150 °C), static extraction time (5 and 10 min) and SS ratio (20, 30, and 40 mL/g).

For the extraction, an appropriate amount of sample was mixed with 2 g of diatomaceous earth, placed into a 34 mL stainless steel extraction cell fitted with two cellulose filters (Dionex™ 350/150 Extraction Cell Filters, Thermo Fisher Scientific Inc.) and extracted according to the conditions of the experimental plan. Thus, the obtained extracts were collected in 250 mL glass vials, transferred and made up to volume in 50 mL volumetric flasks and stored at −18 °C in a nitrogen gas atmosphere until analyzed.

### 3.4. TPC Determination

The TPC of the blackcurrant and bilberry leaves extracts was determined using the Folin–Ciocalteu spectrophotometric method described by Shortle et al. [67] with some modifications. A volume of 100 µL of each extract was mixed with 200 µL of Folin–Ciocalteu reagent, 2 mL distilled water and, after 3 min, 1 mL of a 20% sodium carbonate solution. The reaction mixtures were thermostated at 50 °C for 25 min, and afterwards the absorbance was measured at 765 nm. A blank was prepared with the extraction solvent instead of the extract, using the same reagents. TPC is expressed as mg gallic acid equivalents (GAE) per g of leaves dw according to the gallic acid calibration curve.

### 3.5. UPLC ESI MS^2^ Analysis

The identification and quantification of individual phenolic compounds in the optimized MAE and PLE extracts of blackcurrant and bilberry leaves was performed by UPLC ESI MS^2^ analysis on an Agilent 1290 series RRLC instrument (Agilent, Santa Clara, CA, USA) equipped with a triple quadrupole mass spectrometer (6430) with an ESI ion source. ESI ionization was conducted in both positive and negative modes (*m*/*z* 100 to 1000) with nitrogen (99.999%, Messer, Zagreb, Croatia) as the induction cone and collision gas under the following conditions: positive/negative capillary voltage 4000/3500 V, drying gas temperature 300 °C, flow rate 11 L/h, and nebulizer pressure 40 psi. The chromatographic separations were performed on a Zorbax Eclipse Plus C18 column (Agilent, 100 × 2.1 mm; 1.8 µm particle size) at 35 °C with an injection volume of 2.5 µL. The data were collected and processed by Agilent MassHunter workstation software (ver. B.04.01). The used method and solvent composition, as well as the quality parameters, including calibration curves and instrumental limits of detection (LOD) and quantification (LOQ), were previously described by Elez Garofulić et al. (2018) [38]. Identification and quantitative determination were performed using the calibration curves of the following standards: gallic, chlorogenic, caffeic, *p*-coumaric, syringic, rosmarinic and ferulic acids, quercetin-3-glucoside, quercetin-3-rutinoside, kaempferol-3-rutinoside, myricetin, catechin, epicatechin, epigallocatechin gallate, epicatechin gallate, luteolin, apigenin, and procyanidin B2. For compounds lacking commercial reference standards, a tentative identification was performed according to the mass spectral data and literature reports of their mass fragmentation patterns. Quantification was performed as follows: the amounts of quinic and 3,5-dicaffeoylquinic acid were calculated according to the chlorogenic acid calibration curve, those of *p*-hydroxybenzoic acid, 3,4-dihydroxybenzoic acid hexoside and galloylquinic acid according to the gallic acid calibration curve, those of 3-*p*-cumaroylquinic acid according to the *p*-coumaric acid calibration curve, those of ferulloylquinic acid according to the ferulic acid calibration curve, those of quercetin, quercetin dihexoside, quercetin pentosylhexoside, quercetin acetylhexoside, quercetin glucuronide, quercetin rhamnoside, quercetin pentoside, isorhamnetin pentosylhexoside and isorhamnetin hexoside according to the quercetin 3-glucoside calibration curve, those of isorhamnetin rutinoside according to the quercetin-rutinoside calibration curve, those of kaempferol acetylrutinoside, kaempferol pentosylhexoside, kaempferol pentoside, kaempferol acetylhexoside, kaempferol deoxyhexoside and kaempferol according to the kaempferol-3-glucoside calibration curve, those of myricetin galactoside, myricetin rhamnoside and myricetin arabinoside according to the myricetin calibration curve, those of apigenin deoxyhexosyl hexoside according to the apigenin calibration curve, those of luteolin rutinoside and luteolin glucoside according to the luteolin calibration curve, and those of procyanidin B1 and procyanidin trimer according to the procyanidin B2 calibration curve. All results are expressed in mg/g dw.

### 3.6. Antioxidant Capacity Determination

#### 3.6.1. Ferric Reducing Antioxidant Power (FRAP) Assay

The FRAP assay was performed according to the methodology of Benzie and Strain [68], with some modifications. First, the FRAP reagent was prepared by mixing a sodium acetate buffer (0.3 M, pH 3.6), a 0.01 M TPTZ solution in 0.04 M hydrochloric acid and a 20 mM FeCl_3_ 6H_2_O aqueous solution in the ratio of 10:1:1. An aliquot of 80 µL of the extract was mixed with 240 µL of distilled water and 2080 µL of the FRAP reagent. The mixture was shaken at 1800 rpm on a Vortex MS2 Minishaker IKA (IKA, Staufen, Germany) and thermostated at 37 °C for 5 min. A blank experiment was performed with the extraction solvent instead of the extract and with the same reagents. Absorbance was measured at 593 nm, and the results are expressed as µmol TE/g dw according to the Trolox standard calibration curve.

#### 3.6.2. 2,2-Diphenyl-1-picrylhydrazyl Radical (DPPH) Scavenging Assay

The DPPH assay was performed according to the method of Braca et al. [69]. For the analysis, 0.75 mL of the extract was mixed with 1.5 mL of a 0.2 mM DPPH solution. The mixture was kept at room temperature in the dark for 20 min. Then, the absorbance was measured at 517 nm against 100% methanol as a blank. The results are expressed as µmol TE/g dw according to the Trolox standard calibration curve.

#### 3.6.3. 2,2-Azinobis(3-ethylbenzothiazoline-6-sulfonic Acid (ABTS) Assay

The antioxidant capacity of ABTS was measured according to a modified method described by Miller and Rice-Evans [70]. An amount of 40 µL of the appropriately diluted sample was mixed with 4 mL of 1% ABTS•+, and the absorbance was measured at 734 nm after 1 min. The results are expressed as µmol TE/g dw according to the Trolox standard calibration curve.

#### 3.6.4. Oxygen Radical Absorbance Capacity (ORAC) Assay

The ORAC assay was performed according to a previously reported method [53,71], with some modifications. For the analysis, an automated plate reader (BMG LABTECH, Offenburg, Germany) with 96-well plates was used, and the data were analyzed by MARS 2.0 software. The diluted samples were placed in a black plate containing a fluorescein solution (70.3 nM) and incubated for 30 min at 37 °C. After the first three cycles (representing the baseline signal), 240 mM AAPH was injected into each well to initiate the peroxyl radical generation. Different dilutions of Trolox (3.12–103.99 µM) were used in each plate as a reference standard. Fluorescence intensity (excitation at 485 nm and emission at 528 nm) was monitored every 90 s over a total measurement period of 120 min, and the results are expressed as µmol TE/g dw according to the Trolox standard calibration curve.

### 3.7. Statistical Analysis

Statistical analysis was performed by Statistica ver. 10.0 (Statsoft Inc., Tulsa, OK, USA) software. For the evaluation of the MAE and PLE extraction techniques, a mixed 2- and 3-level full factorial design was employed, comprising 18 experimental trials. The observed factors were temperature, extraction time and SS ratio, while the dependent response was the TPC of the blackcurrant and bilberry leaves. All extractions and analyses were performed in duplicate. For the assessment of the basic information about the experimental data set, descriptive statistics was employed, and the data are presented as mean values ± SE. The obtained data and residuals were checked for their normality and homoscedasticity by the Shapiro–Wilk and Levene’s tests, respectively, and were accordingly analyzed by ANOVA coupled with the post-hoc Tukey’s HSD test or by the non-parametric Kruskal–Wallis test coupled with multiple comparison of mean ranks. Differences between individual polyphenolic composition and antioxidant capacity of the optimized MAE and PLE extracts were tested by one-way ANOVA followed by the post-hoc Tukey’s HSD test. The relationships between the determined polyphenols and the antioxidant capacity were tested by calculating the Pearson’s correlation coefficients. All tests were considered significant at *p* ≤ 0.05.

## 4. Conclusions

Although underestimated and often discarded during fruit harvesting, blackcurrant and bilberry leaves represent a valuable source of polyphenols. Through the employment of green extraction techniques such as MAE and PLE, the utilization of leaf polyphenols meets the requirements of sustainable production according to the zero-waste concept. Optimizing the extraction parameters maximized the process output with regard to the specific properties of the blackcurrant and bilberry leaves and their polyphenolic composition. When MAE was applied, both blackcurrant and bilberry leaves provided the highest polyphenolic yield at 80 °C and with the highest SS ratio (40 mL/g), differing only with regard to the extraction time, as the bilberry polyphenols required a longer extraction time than the 5 min that were sufficient for the polyphenols in blackcurrant leaves. In the case of PLE, the established optimal conditions were 150 °C/5 min/30 mL/g for blackcurrant and 125 °C/10 min/40 mL/g for bilberry leaves. The obtained extracts were most rich in flavonols and their glycosides, followed by phenolic acids and procyanidins. Despite their lower contribution to the polyphenolic composition of the extracts, flavan-3-ols and procyanidins were found to be the compounds most responsible for the high antioxidant capacity of the extracts, based on the very high positive correlation with antioxidant capacity in the ABTS and ORAC assays. In terms of both total polyphenolic content and concentration of most of the individual compounds observed, as well as of the antioxidant capacity of the extracts obtained, PLE stood out as more effective than MAE for isolating polyphenols from blackcurrant and bilberry leaves, providing valuable extracts for future applications in the food and nutraceutical industry.

## Figures and Tables

**Table 1:** TPC of blackcurrant and bilberry leaves extracts obtained under different MAE and PLE conditions.

Extraction Technique	Extraction Parameters			TPC mg/g dw	
	Temperature (°C)	Time (min)	SS Ratio (mL/g)	Blackcurrant	Bilberry
MAE	60	5	20	49.45 ± 1.28	41.21 ± 0.27
			30	58.54 ± 0.73	34.32 ± 0.06
			40	60.88 ± 2.45	39.56 ± 3.03
		10	20	53.06 ± 0.06	37.41 ± 1.15
			30	53.76 ± 2.44	43.37 ± 3.10
			40	63.07 ± 0.32	46.71 ± 0.55
	70	5	20	51.40 ± 2.09	40.79 ± 1.11
			30	53.58 ± 2.24	43.01 ± 0.11
			40	61.02 ± 0.39	50.73 ± 2.16
		10	20	50.70 ± 1.82	45.35 ± 2.87
			30	54.27 ± 1.97	47.28 ± 0.10
			40	57.78 ± 0.04	64.19 ± 0.58
	80	5	20	54.96 ± 0.12	44.73 ± 2.44
			30	56.47 ± 0.17	52.84 ± 0.04
			40	62.10 ± 3.25	56.20 ± 2.47
		10	20	53.38 ± 1.01	45.55 ± 0.23
			30	56.34 ± 1.37	45.09 ± 0.57
			40	73.26 ± 2.28	56.06 ± 0.15
PLE	100	5	20	53.07 ± 0.06	33.74 ± 0.07
			30	58.85 ± 0.70	65.88 ± 0.46
			40	54.55 ± 0.59	59.53 ± 0.41
		10	20	52.94 ± 0.21	49.69 ± 0.95
			30	42.92 ± 0.04	57.75 ± 0.79
			40	52.76 ± 0.12	51.07 ± 0.52
	125	5	20	60.52 ± 0.59	70.54 ± 0.07
			30	67.62 ± 1.58	54.62 ± 0.35
			40	62.15 ± 0.13	45.50 ± 0.26
		10	20	71.02 ± 3.35	60.30 ± 0.35
			30	70.84 ± 5.24	53.78 ± 0.55
			40	66.70 ± 5.59	70.55 ± 1.40
	150	5	20	69.95 ± 0.58	58.66 ± 0.95
			30	78.90 ± 0.02	53.78 ± 0.55
			40	62.23 ± 0.75	55.80 ± 0.21
		10	20	68.58 ± 2.60	56.41 ± 1.24
			30	69.49 ± 1.52	54.85 ± 0.18
			40	69.88 ± 2.01	62.70 ± 1.56

TPC—total phenolic content, MAE—microwave-assisted extraction, PLE—pressurized liquid extraction. Results are expressed as mean ± standard deviation.

**Table 2:** Statistical analysis of the results for the effect of MAE and PLE parameters (temperature, time and SS ratio) on the TPC of blackcurrant and bilberry leaves.

MAE	TPC (mg/g dw)		PLE	TPC (mg/g dw)	
	Blackcurrant	Bilberry		Blackcurrant	Bilberry
Temperature (°C)	*p* = 0.52	*p* < 0.01 *	Temperature (°C)	*p* < 0.01 *	*p* = 0.44
60	56.46 ± 1.47 ^a^	40.43 ± 1.27 ^a^	100	52.51 ± 1.44 ^a^	52.94 ± 3.06 ^a^
70	54.79 ± 1.14 ^a^	48.56 ± 2.33 ^b^	125	66.47 ± 1.41 ^b^	59.21 ± 2.75 ^a^
80	57.75 ± 2.54 ^a^	50.08 ± 1.56 ^b^	150	69.84 ± 1.50 ^b^	57.03 ± 0.91 ^a^
Time (min)	*p* = 0.88	*p* = 0.17	Time (min)	*p* = 0.73	*p* = 0.80
5	56.49 ± 1.07 ^a^	44.82 ± 1.64 ^a^	5	63.09 ± 1.84 ^a^	55.34 ± 2.48 ^a^
10	56.18 ± 1.82 ^a^	47.89 ± 1.80 ^a^	10	62.79 ± 2.44 ^a^	57.45 ± 1.48 ^a^
SS ratio (mL/g)	*p* < 0.01 *	*p* < 0.01 *	SS ratio (mL/g)	*p* = 0.66	*p* = 0.83
20	52.16 ± 0.62 ^a^	42.51 ± 0.96 ^a^	20	62.68 ± 2.33 ^a^	54.89 ± 3.41 ^a^
30	53.83 ± 1.20 ^a^	44.32 ± 1.69 ^a^	30	64.77 ± 3.47 ^a^	56.78 ± 1.30 ^a^
40	63.02 ± 1.52 ^b^	52.24 ± 2.39 ^b^	40	61.38 ± 1.91 ^a^	57.53 ± 2.44 ^a^

TPC—total phenolic content, MAE—microwave-assisted extraction, PLE—pressurized liquid extraction. Results are expressed as mean value ± standard error. * Statistically significant variable at *p* ≤ 0.05. Means with the same letter within the column are not significantly different at *p* ≤ 0.05.

**Table 3:** Individual polyphenols obtained from blackcurrant leaves under optimized MAE and PLE conditions.

Blackcurrant Leaves Extracts’ Individual Polyphenols	Precursor Ion (*m/z*)	Product Ion (*m/z*)	MAE (mg/g dw)	PLE (mg/g dw)
Phenolic acids				
Quinic acid	191	85	0.02 ± 0.00 ^a^	0.06 ± 0.00 ^b^
Ferulic acid *	193	134	0.02 ± 0.00 ^a^	0.02 ± 0.00 ^a^
3-*p*-Coumaroylquinic acid	337	163	0.01 ± 0.00 ^a^	0.13 ± 0.00 ^b^
Rosmarinic acid *	359.1	161	0.02 ± 0.00 ^a^	0.02 ± 0.00 ^a^
Chlorogenic acid *	353	191	0.40 ± 0.02 ^a^	0.88 ± 0.02 ^b^
3,5-Dicaffeoylquinic acid	515	191	-	0.05 ± 0.00
Syringic acid *	197	182	0.10 ± 0.0 ^a^	0.14 ± 0.00 ^b^
Caffeic acid *	179	135	0.62 ± 0.0 ^a^	0.59 ± 0.00 ^a^
Galloylquinic acid	343	191	0.02 ± 0.00 ^a^	0.04 ± 0.00 ^b^
Ferruloylquinic acid	367	193	-	0.03 ± 0.00
Gallic acid *	169	125	0.07 ± 0.00 ^a^	0.25 ± 0.00 ^b^
*p*-Coumaric acid *	163	119	0.04 ± 0.00 ^a^	0.08 ± 0.00 ^b^
*p*-Hydroxybenzoic acid	137	93	0.39 ± 0.01 ^b^	0.19 ± 0.00 ^a^
3,4-Dihydroxybenzoic acid hexoside	317	155	-	0.01 ± 0.00
Flavonols				
Isorhamnetin rutinoside	625	317	0.01 ± 0.00 ^a^	0.13 ± 0.01 ^b^
Isorhamnetin hexoside	479	317	0.01 ± 0.00 ^a^	0.18 ± 0.01 ^b^
Kaempferol-3-glucoside	449	287	0.03 ± 0.00 ^a^	1.75 ± 0.07 ^b^
Myricetin *	319	273	0.33 ± 0.01 ^b^	0.24 ± 0.00 ^a^
Quercetin glucuronide	479	303	0.02 ± 0.00 ^a^	0.02 ± 0.00 ^a^
Quercetin rhamnoside	449	303	0.02 ± 0.00 ^a^	0.04 ± 0.00 ^b^
Quercetin pentoside	435	303	-	0.06 ± 0.00
Myricetin rhamnoside	465	319	0.06 ± 0.00 ^b^	0.05 ± 0.00 ^a^
Kaempferol pentoside	419	287	-	0.02 ± 0.00
Quercetin acetylhexoside	507	303	0.01 ± 0.00 ^a^	1.82 ± 0.06 ^b^
Kaempferol acetylhexoside	491	287	-	0.36 ± 0.01
Myricetin galactoside	481	319	0.05 ± 0.00 ^a^	0.69 ± 0.02 ^b^
Quercetin-3-glucoside *	465	303	0.07 ± 0.00 ^a^	3.11 ± 0.09 ^b^
Myricetin arabinoside	451	319	0.20 ± 0.01 ^b^	0.09 ± 0.00 ^a^
Isorhamnetin dihexoside	641	317	0.02 ± 0.00 ^a^	0.22 ± 0.01 ^b^
Quercetin-3-rutinoside *	611	303	0.05 ± 0.00 ^a^	4.46 ± 0.21 ^b^
Isorhamnetin pentosylhexoside	511	317	0.01 ± 0.00 ^a^	0.06 ± 0.00 ^b^
Kaempferol-3-rutinoside *	595	287	0.01 ± 0.00 ^a^	0.65 ± 0.01 ^b^
Quercetin	303	303	3.68 ± 0.12 ^b^	2.80 ± 0.11 ^a^
Kaempferol	287	287	3.38 ± 0.08 ^a^	4.77 ± 0.16 ^b^
Quercetin pentosylhexoside	597	303	-	0.02 ± 0.00
Flavan-3-ols				
Catechin *	291	139	0.28 ± 0.01 ^a^	0.83 ± 0.02 ^b^
Epicatechin *	291	139	0.12 ± 0.00 ^a^	0.87 ± 0.05 ^b^
Epigallocatechingallate *	459	289	0.05 ± 0.00 ^b^	0.03 ± 0.00 ^a^
Epicatechingallate *	423	273	0.08 ± 0.00 ^a^	0.07 ± 0.00 ^a^
Flavones				
Luteolin *	287	153	0.07 ± 0.00 ^a^	0.25 ± 0.01 ^b^
Apigenin *	271	153	0.02 ± 0.00	-
Luteolin glucoside	449	287	0.03 ± 0.00	-
Apigenin deoxyhexosyl hexoside	579	459	-	0.04 ± 0.00
Luteolin rutinoside	595	287	-	0.64 ± 0.03
Procyanidins				
Procyanidin trimer	865	575	-	0.29 ± 0.01
Procyanidin B1	579	291	0.83 ± 0.03 ^a^	2.52 ± 0.11 ^b^
Procyanidin B2 *	579	291	0.02 ± 0.00	-
Total UPLC ESI MS^2^-identified polyphenols			11.21 ± 0.21 ^a^	29.59 ± 0.73 ^b^

MAE—microwave-assisted extraction, PLE—pressurized liquid extraction. * Identified by authentic standards. Values are expressed as mean ± standard deviation. Values with the same letter within a row are not significantly different at *p* ≤ 0.05.

**Table 4:** Individual polyphenols obtained from bilberry leaves under optimized MAE and PLE conditions.

Bilberry Leaves Extracts’ Individual Polyphenols	Precursor Ion (*m/z*)	Product Ion (*m/z*)	MAE (mg/g dw)	PLE (mg/g dw)
Phenolic acids				
Ferulic acid *	193	134	-	0.03 ± 0.00
Rosmarinic acid *	359.1	161	-	0.02 ± 0.00
Chlorogenic acid *	353	191	0.12 ± 0.00 ^a^	0.24 ± 0.01 ^b^
3,5-Dicaffeoylquinic acid	515	191	-	0.02 ± 0.00
Syringic acid *	197	182	0.17 ± 0.00 ^b^	0.10 ± 0.00 ^a^
Caffeic acid *	179	135	0.04 ± 0.00 ^a^	0.80 ± 0.05 ^b^
Gallic acid *	169	125	0.12 ± 0.00 ^a^	0.19 ± 0.01 ^b^
*p*-Coumaric acid *	163	119	0.03 ± 0.00 ^b^	0.02 ± 0.00 ^a^
*p*-Hydroxybenzoic acid	137	93	0.07 ± 0.00 ^a^	0.09 ± 0.00 ^b^
3,5-Digalloylquinic acid	495	343	-	0.01 ± 0.00
Flavonols				
Isorhamnetin rutinoside	625	317	-	0.14 ± 0.00
Isorhamnetin hexoside	479	317	-	0.15 ± 0.01
Kaempferol-3-glucoside	449	287	-	1.19 ± 0.06
Myricetin *	319	273	0.12 ± 0.00 ^a^	0.14 ± 0.00 ^b^
Quercetin glucuronide	479	303	0.03 ± 0.00 ^a^	0.08 ± 0.00 ^b^
Kaempferol glucuronide	463	287	-	0.01 ± 0.00
Quercetin rhamnoside	449	303	0.05 ± 0.00 ^a^	0.12 ± 0.00 ^b^
Kaempferol deoxyhexoside	434	287	-	0.01 ± 0.00
Quercetin pentoside	435	303	-	0.11 ± 0.00
Myricetin rhamnoside	465	319	0.02 ± 0.00 ^a^	0.10 ± 0.00 ^b^
Kaempferol pentoside	419	287	-	0.05 ± 0.00
Kaempferol pentosylhexoside	581	287	-	0.02 ± 0.00
Quercetin acetylhexoside	507	303	0.01 ± 0.00 ^a^	0.05 ± 0.00 ^b^
Kaempferol acetylhexoside	491	287	0.01 ± 0.00 ^a^	0.03 ± 0.00 ^b^
Myricetin galactoside	481	319	0.07 ± 0.00 ^a^	2.88 ± 0.08 ^b^
Quercetin-3-glucoside *	465	303	0.02 ± 0.00 ^a^	7.43 ± 0.28 ^b^
Myricetin arabinoside	451	319	0.21 ± 0.00 ^b^	0.11 ± 0.01 ^a^
Quercetin acetylrutinoside	653	303	-	0.01 ± 0.00
Kaempferol acetylrutinoside	637	287	0.01 ± 0.00	-
Quercetin dihexoside	625	317	0.02 ± 0.00 ^a^	0.03 ± 0.00 ^b^
Quercetin-3-rutinoside *	611	303	0.03 ± 0.00 ^a^	1.08 ± 0.06 ^b^
Isorhamnetin pentosylhexoside	611	317	-	0.02 ± 0.00
Kaempferol-3-rutinoside *	595	287	0.01 ± 0.00 ^a^	0.81 ± 0.03 ^b^
Quercetin *	303	303	2.03 ± 0.10 ^a^	5.27 ± 0.14 ^b^
Kaempferol *	287	287	2.40 ± 0.12 ^a^	4.35 ± 0.13 ^b^
Quercetin pentosylhexoside	597	303	0.01 ± 0.00	-
Flavan-3-ols				
Catechin *	291	139	0.14 ± 0.00 ^b^	0.07 ± 0.00 ^a^
Epicatechin *	291	139	0.04 ± 0.00 ^a^	0.08 ± 0.00 ^b^
Epigallocatechingallate *	459	289	0.03 ± 0.00 ^a^	0.08 ± 0.00 ^b^
Epicatechingallate *	423	273	0.08 ± 0.00 ^a^	0.10 ± 0.00 ^b^
Flavones				
Luteolin *	287	153	0.04 ± 0.00 ^a^	0.63 ± 0.04 ^b^
Apigenin *	271	153	0.02 ± 0.00	-
Luteolin rutinoside	595	287	-	0.79 ± 0.04
Procyanidins				
Procyanidin trimer	865	575	0.01 ± 0.00 ^a^	0.05 ± 0.00 ^b^
Procyanidin B1	579	291	1.07 ± 0.04 ^b^	0.50 ± 0.03 ^a^
Total UPLC ESI MS^2^-identified polyphenols			7.01 ± 0.13 ^a^	27.99 ± 0.39 ^b^

MAE—microwave-assisted extraction, PLE—pressurized liquid extraction. * Identified by authentic standards. Values are expressed as mean ± standard deviation. Values with the same letter within a row are not significantly different at *p* ≤ 0.05.

**Table 5:** Antioxidant capacity of the optimized MAE and PLE extracts of blackcurrant and bilberry leaves obtained by FRAP, DPPH, ABTS and ORAC assays.

Extract		Antioxidant Capacity (µmol TE/g dw)			
		FRAP	DPPH	ABTS	ORAC
		*p* < 0.01 *	*p* < 0.01 *	*p* < 0.01 *	*p* < 0.01 *
Blackcurrant	MAE	623.53 ± 26.04 ^a^	534.85 ± 3.46 ^b^	648.04 ± 8.97 ^a^	320.82 ± 5.58 ^a^
	PLE	934.07 ± 24.01 ^b^	495.98 ± 0.90 ^a^	1149.35 ± 40.55 ^b^	441.48 ± 8.74 ^b^
		*p* < 0.01 *	*p* < 0.01 *	*p* = 0.01 *	*p* = 0.85
Bilberry	MAE	263.33 ± 12.57 ^a^	314.72 ± 1.06 ^a^	564.94 ± 11.32 ^a^	327.73 ± 6.34 ^a^
	PLE	471.31 ± 15.85 ^b^	388.76 ± 9.80 ^b^	659.00 ± 11.10 ^b^	332.48 ± 7.06 ^a^

MAE—microwave-assisted extraction, PLE—pressurized liquid extraction. Results are expressed as mean value ± standard error. * Statistically significant variable at *p* ≤ 0.05. Means with the same letter within a column are not significantly different at *p* ≤ 0.05.

**Table 6:** Pearson’s correlation coefficients for blackcurrant and bilberry leaves’ polyphenols and antioxidant capacity in optimized MAE and PLE extracts.

Polyphenols	FRAP	DPPH	ABTS	ORAC
TPC	0.48	0.46	0.87	0.82
Total UPLC ESI MS^2^	0.19	0.26	0.70	0.66
Phenolic acids	0.68	0.81	0.86	0.75
Flavonols	0.02	0.16	0.57	0.53
Flavan-3-ols	0.90	0.54	0.99 *	0.98 *
Flavones	−0.19	0.00	0.39	0.36
Procyanidins	0.91	0.36	0.94	0.96 *

TPC—total phenolic content. * Statistically significant at *p* ≤ 0.05.

## Data Availability

The original contributions presented in the study are included in the article; further inquiries can be directed to the corresponding author.
